# Elevated psychiatric and substance use disorders in people with intellectual disabilities in a Swedish National Sample

**DOI:** 10.1017/S0033291725102882

**Published:** 2025-12-29

**Authors:** Ruth C Brown, Linda Abrahamsson, Jan Sundquist, Kristina Sundquist, Kenneth Kendler

**Affiliations:** 1 Department of Psychiatry, Virginia Commonwealth University, USA; 2 Center for Primary Health Care Research, Lund University, Sweden

**Keywords:** epidemiology, health disparities, intellectual disability, population study, psychiatric disorders, registry data, substance use disorders

## Abstract

**Background:**

Adults with intellectual disability (ID) experience marked mental health inequities, yet population-wide estimates that capture both primary- and specialist-care diagnoses remain scarce.

**Methods:**

Using nationwide Swedish registries, including primary care, specialist, inpatient, prescription drug, criminal, and suspicion registers, we included all individuals born in Sweden between 1958 and 1997 (N = 3,970,600), including 38,818 individuals with ID diagnoses (0.98%; 49.1% mild, 13.3% moderate, and 9.6% severe/profound). Hazard ratios (HR) were calculated using Cox proportional hazards regression to estimate the relative risk of lifetime diagnoses of major depression, anxiety disorders (ANX), obsessive-compulsive disorder (OCD), bipolar disorder (BD), attention-deficit/hyperactivity disorder (ADHD), drug use disorder, alcohol use disorder, schizophrenia, and other nonaffective psychosis. Additionally, cohort effects on psychiatric diagnosis risks in adults with intellectual disabilities versus the general population were evaluated.

**Results:**

People with ID were at higher risk for all psychiatric and substance use disorders, with HRs ranging from 1.7–2.0 for major depression and anxiety, drug and alcohol use disorders, 3.5–5.8 for BD, OCD and ADHD, and 10.9–12.7 for schizophrenia and other nonaffective psychosis. Higher prevalence was consistently seen among those with mild versus moderate or severe/profound intellectual disability. Relative risks narrowed modestly in successive birth cohorts, although absolute differences remained substantial.

**Conclusions:**

Across six decades of follow-up, adults with ID faced markedly higher psychiatric and substance-use morbidity – most pronounced for psychotic disorders – than the general population. Whole-system mental-health screening and tailored interventions are required to address this persistent disparity.

## Introduction

Psychiatric care is increasingly moving toward precision population health; however, adults with intellectual disability (ID) remain underrepresented in psychiatric research and clinical practice (Burke & Heller, 2017; Krahn & Fox, 2014). ID is a developmental disorder affecting approximately 1% of the global population and is characterized by significant limitations in intellectual and adaptive functioning (American Psychiatric Association, 2013; Maulik et al., 2011; McKenzie et al., 2016). People with ID have been recognized as a health disparity population (National Institutes of Health, 2023), experience markedly elevated morbidity and mortality compared to the general population, and in turn, experience higher rates of mortality, decreased life expectancy, increased health care costs, and poorer quality of life (Cooper et al., 2015; Hirvikoski et al., 2021; Kinnear et al., 2018). However, psychiatrists frequently report inadequate training, insufficient knowledge, and low confidence in managing psychiatric disorders in individuals with ID, often resulting in reluctance or discomfort when treating this population (Fredheim et al., 2013; Pelleboer-Gunnink, Oorsouw, Weeghel, & Embregts, 2017; Werner, Stawski, Polakiewicz, & Levav, 2013).

Seminal studies, including both primary research and systematic reviews, have substantially advanced our understanding of the risk for psychiatric disorders people with ID (Cooper & Bailey, 2001; Deb, Thomas, & Bright, 2001; Emerson, 2003; Rutter et al., 1976). Despite these important contributions from primary research, systematic reviews and meta-analyses synthesizing evidence across multiple populations have documented considerable methodological heterogeneity and inconsistent prevalence estimates(Mazza, Rossetti, Crespi, & Clerici, 2020; Smiley, 2005; Yoo, Valdovinos, & Schroeder, 2012), making it challenging to determine precise prevalence rates.

Findings regarding the relationship between ID severity and psychiatric risk remain inconsistent. Regional population-based cohort studies have reported higher risks in severe/profound ID compared to mild or moderate ID (Cooper & Bailey, 2001; Cooper, Smiley, Morrison, Williamson et al., 2007), while meta-analytic evidence suggests elevated risks among individuals with mild/moderate ID compared to severe or profound ID (Mazza et al., 2020). Evidence from both primary research and systematic reviews indicates that schizophrenia and psychosis are consistently elevated among people with ID compared to the general population. This pattern has been documented in meta-analyses (Aman, Naeem, Farooq, & Ayub, 2016), regional population-based cohorts(Cooper, Smiley, Morrison, Allan et al., 2007), psychiatric and ID registry linkages (Morgan, Leonard, Bourke, & Jablensky, 2008), and nationally representative surveys (White et al., 2005), supporting theories of shared neurodevelopmental origins (Murray, Bhavsar, Tripoli, & Howes, 2017; Owen & O’Donovan, 2017; Owen, O’Donovan, Thapar, & Craddock, 2011).

Conversely, data on substance use disorders among people with ID remain sparse and inconclusive, primarily focusing on substance use rather than clinically significant misuse (Hassiotis et al., 2008, 2011; Huxley, Dalton, Tsui, & Hayhurst, 2019; Mcgillivray & Moore, 2001; McGuire, Daly, & Smyth, 2007; Robertson et al., 2000). Narrative and systematic reviews suggest that alcohol and substance use among people with ID may be increasing as a result of deinstitutionalization and community integration (Bhatt & Gentile, 2021; Huxley et al., 2019), highlighting an emerging area of public health concern. Nevertheless, evidence to date is largely derived from small sample sizes or specialist-care registries, leaving a critical gap in knowledge needed to guide clinical services and policy for this vulnerable group in community practices (Mazza et al., 2020; Wolstencroft et al., 2022; Yoo et al., 2012).

Whole population-based national registries provide more precise and representative estimates of the relative risk of psychiatric disorders than other sampling methods typically employed in the ID literature (e.g. clinic-based or county-level sampling). Such investigations are rare for the ID population. Prior population-based research, such as studies by Axmon, Björne, Nylander, and Ahlström (2018a, 2018b) and Axmon, Ahlström, Gagnemo Persson, & Eberhard (2019), examined the prevalence of psychiatric diagnoses in older Swedish adults (aged 55+ in 2012), using the national disability services registry (LSS) and the National Patient Register, which includes both inpatient and outpatient specialist service recipients. This research revealed increased risks for most psychiatric disorders, particularly psychotic, affective, and anxiety disorders, compared to the general population, with a lower risk for substance use disorders and a narrowing gap between the oldest and youngest cohorts (Axmon et al., 2018a). However, studies have found that a substantial portion of psychiatric cases are identified and managed solely in primary care settings in Sweden (Sundquist, Ohlsson, Sundquist, & Kendler, 2017),and worldwide (Wang et al., 2007); similarly, high rates of primary care mental health prescribing has been found for people with ID in the UK (Sheehan et al., 2015). Thus, identifying patterns of psychiatric care utilization in primary care among people with ID is vital. Further investigation into younger cohorts who have come of age in the era of deinstitutionalization and increased community inclusion is necessary (Tøssebro, 2016).

Here we investigate major depression (MD), anxiety disorders (ANX), obsessive-compulsive disorder (OCD), bipolar disorder (BP), attention-deficit hyperactivity (ADHD), drug use disorder (DUD), alcohol use disorder (AUD), schizophrenia (SZ), and other nonaffective psychosis (ONAP). We examine the following aims: (1) describe the lifetime prevalence of common psychiatric disorders in adults born between 1958 and 1997 with ID, stratified by ID severity, in the Swedish population across multiple registries (in particular Hospital-based, Outpatient, versus Primary Care) compared to the general population (G-Pop), (2) estimate the risk for each psychiatric and substance use disorders for adults with ID compared to the G-Pop, (3) identify the extent of psychiatric primary care utilization among people with ID and co-occurring psychiatric disorders compared to G-Pop, and (4) explore the impact of birth cohort on the risk for psychiatric diagnoses in adults with ID compared to the G-Pop. To the best of our knowledge, this study is the most comprehensive national, longitudinal cohort study of people with ID.

## Methods

Data for this study were collected from the nationwide Swedish registers (see Supplementary Table 1 for details). Unique identification numbers, replaced with a serial number for confidentiality, were used for registry linkage. Ethical approval was secured from the regional ethical review board in Lund, Sweden (No. 2008/409 and later amendments). Our study population (n = 3,970,600) consisted of all individuals born in Sweden between 1958 and 1997, alive and living in Sweden until both age 18 and year 2000, to ensure enough follow-up time for retrieving psychiatric diagnoses from the registries. ID diagnoses and psychiatric diagnoses of MD, ANX, OCD, BD, ADHD, DUD, AUD, SZ, and ONAP were searched for using the Swedish Hospital Discharge Register, Outpatient Care Register, almost nationwide primary care data, the Swedish Prescribed Drug Register, the Swedish Cause of Death Register, the Swedish Criminal Register, and the Swedish Suspicion Register. ID diagnoses were based on standard ICD codes (ICD-10: F70-F79; ICD-9: 317–319), with severity classified as mild, moderate, or severe/profound. For details on how all diagnoses were defined, see Supplementary Table 2. Psychiatric diagnoses other than ID prior to age five were censored to improve diagnostic validity. As a proxy for socioeconomic status, we used parental educational level (pre-secondary, secondary, or post-secondary). Education levels were retrieved from the LISA and the Population and Housing Censuses, with linkage to biological parents via the Multi-Generation Register, described in Supplementary Table 1.

We used multivariate Cox proportional hazards regression models to examine the effect of lifetime ID diagnoses on time to first diagnosis of MD, ANX, OCD, BD, ADHD, DUD, AUD, SZ, and ONAP, using age as a time scale. Individuals were followed from age five to first diagnosis, emigration, death, or study end of 2018-12-31, whichever occurred first. Models were controlled for sex, birth year, parental educational, and parental birth year, with interaction effects between age and these covariates to adjust for non-proportional hazards. The interaction effects with age were assessed by splitting data over time. Two more detailed sets of analyses were performed. First, to examine differences in psychiatric disorder outcomes by ID severity, we estimated interaction effects between ID and severity within the same modeling framework, coding severity with dummy variables for mild, moderate, severe, and unspecified ID. Second, to evaluate differences across birth cohorts, models were stratified on the four groups: 1958–1967, 1968–1977, 1978–1987, and 1988–1997. In the full sample, we also included an interaction term between ID and birth year (on a scale of 10 years) to test for linear trends of year of birth on the effect of ID on the outcomes. Data analysis was conducted from August 22, 2023 to September 25, 2024, using R, version 4.4.0 (R Core Team, 2024) (Supplementary Table 3) and SAS, version 9.4 (SAS Institute, 2012).

## Results

Registry data identified 38,818 individuals with an ID diagnosis out of the 3,970,600 total population (lifetime prevalence of 0.98%; 0.90% in females, 1.05% in males). Among those with ID, 49.1% (n = 19,051) had mild ID, 13.3% (n = 5,157) had moderate ID, and 9.6% (n = 3,742) had severe/profound ID. The age distribution at the end of follow-up differed substantially between the G-Pop and ID groups, reflecting differences in birth cohort composition. The ID group was younger on average (mean age 36.1 years, SD = 11.5) than the G-Pop (mean age 41.2 years, SD = 12.0), with individuals with ID being born more recently on average (mean birth year 1982.1 versus 1977.0). Notably, 41.3% of individuals with ID were in the youngest age category (19–29 years) compared to 24.4% of the G-Pop, while only 17.2% were in the oldest category (50–62 years) compared to 29.5% of G-Pop. Table 1 show demographic characteristics of the sample, stratified by ID severity. Table 2 shows the lifetime prevalence of major psychiatric disorder cases, stratified by ID severity. Lifetime prevalence of major psychiatric and substance use disorders was significantly higher among people with ID compared to the G-Pop. Within the ID group, those with mild ID consistently had the highest rates, followed by moderate, and severe/profound ID. Compared to the G-Pop, people with severe/profound ID had a higher prevalence of OCD, BD, ADHD, DUD, SZ, and ONAP but lower rates of MD, ANX, and AUD.

**Table 1.** Sample size, year of birth, age, sex, and parental educational level of the study sample

	G-Pop	Total ID	Mild ID	Moderate ID	Severe/Profound ID
Total number	3,970,600	38,818	19,051	5,157	3,742
Year of birth, mean (SD)	1977.0 (12.0)	1982.1 (11.7)	1984.3 (11.0)	1983.4 (11.3)	1980.9 (11.5)
Age at follow-up, mean (SD); % in categories 19–29, 30–39, 40–49, 50–62.	41.2 (12.0); 24.4, 22.6, 23.5, 29.5	36.1 (11.5); 41.3, 25.6, 15.9, 17.2	34.0 (10.8); 49.3, 24.9, 13.1, 12.7	34.8 (11.1); 45.8, 25.6, 14.1, 14.4	36.3 (11.3); 38.3, 27.1, 18.1, 16.5
Female, No. (%)	1,928,036 (48.6)	17,314 (44.6)	8,775 (46.1)	2,149 (41.7)	1,605 (42.9)
Male, No. (%)	2,042,564 (51.4)	21,504 (55.4)	10,276 (53.9)	3,008 (58.3)	2,137 (57.1)
Average parental level of education^a^					
1. Pre-secondary education, No. (%)	591,078 (14.9)	6,686 (17.2)	3,013 (15.8)	784 (15.2)	545 (14.6)
2. Secondary education, No. (%)	1,976,599 (49.8)	21,492 (55.4)	11,136 (58.5)	2,814 (54.6)	1,903 (50.9)
3. Post-secondary education, No. (%)	1,396,559 (35.2)	10,573 (27.2)	4,879 (25.6)	1,550 (30.1)	1,286 (34.4)
Parental year of birth, mean (SD)	1947.9 (12.8)	1952.3 (13.1)	1954.8 (12.4)	1953.4 (12.5)	1950.7 (12.7)

Abbreviations: G-Pop = general population, ID = intellectual disability, MD = major depression, ANX = anxiety disorders, OCD = obsessive-compulsive disorder, BD = bipolar disorders, ADHD = attention-deficit/hyperactivity disorder, DUD = drug use disorder, AUD = alcohol use disorder, SZ = schizophrenia, ONAP = other non-affective psychoses.

a When the average level fell between two categories, a rounding off to the higher educational level was performed.

**Table 2.** Lifetime prevalence of common psychiatric disorders in the Swedish population stratified by level of intellectual disability

	G-Pop	Total ID	Mild ID	Moderate ID	Severe/Profound ID	HR (95%CI) ID versus G-Pop^a^	HR (95%CI) mild versus moderate^a^	HR (95%CI) mild versus severe^a^
Total number	3,970,600	38,818	19,051	5,157	3,742			
MD	17.4	26.5	32.1	21.4	11.7	1.76 (1.72–1.79)^****^	1.58 (1.48–1.69)^****^	3.00 (2.72–3.31)^****^
ANX	16.4	28.1	33.7	24.2	15.3	1.88 (1.84–1.91)^****^	1.45 (1.36–1.54)^****^	2.26 (2.07–2.46)^****^
OCD	1.2	5.3	6.3	5.7	3.8	4.29 (4.11–4.49)^****^	1.07 (0.95–1.22)	1.51 (1.27–1.80)^****^
BD	1.5	4.6	5.5	5.1	3.6	3.49 (3.32–3.66)^****^	1.05 (0.92–1.21)	1.50 (1.25–1.80)^****^
ADHD	3.5	21.0	29.4	17.8	8.0	5.78 (5.65–5.91)^****^	1.67 (1.56–1.79)^****^	3.45 (3.07–3.88)^****^
DUD	5.6	11.8	15.8	8.7	6.5	1.72 (1.67–1.77)^****^	1.87 (1.69–2.07)^****^	2.17 (1.90–2.47)^****^
AUD	4.6	8.0	10.8	5.9	2.2	1.95 (1.88–2.02)^****^	2.06 (1.82–2.33)^****^	5.63 (4.49–7.06)^****^
SZ	0.4	3.6	4.5	4.7	1.2	12.70 (12.01–13.43)^****^	1.09 (0.94–1.26)	4.91 (3.62–6.64)^****^
ONAP	1.1	9.4	11.5	11.7	4.2	10.93 (10.56–11.32)^****^	1.02 (0.93–1.11)	3.19 (2.71–3.77)^****^

*Note:* Cox regression between total ID and G-Pop with age as time scale, adjusted for sex, birth cohort, and parents’ birth years. Interaction effects between age with the controlling variables were included to adjust for non-proportional hazards.

Abbreviations: G-Pop = general population, ID = intellectual disability, MD = major depression, ANX = anxiety disorders, OCD = obsessive-compulsive disorder, BD = bipolar disorders, ADHD = attention-deficit/hyperactivity disorder, DUD = drug use disorder, AUD = alcohol use disorder, SZ = schizophrenia, ONAP = other non-affective psychoses.

a Significance levels for *p*-values: * < .05, ** < .01, *** < .001, **** < .0001.

**Figure 1.**
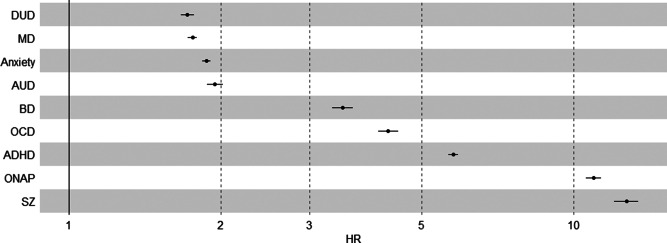
Hazard ratios with 95% confidence intervals of psychiatric risk for people with intellectual disabilities compared to general population. Hazard ratios are adjusted for sex, birth year, parents’ educational levels, and parents’ birth years, with interaction effects between age and each controlling variable.

The hazard ratio was significantly elevated for all disorders (Table 2). The HRs for internalizing and substance use disorders were among the lowest (ranging from HR_DUD_ = 1.72 [1.67–1.77] to HR_AUD_ = 1.95 [1.88–2.02]), whereas disorders previously associated with neurodevelopment – OCD, BD, ADHD, SZ, and ONAP – yielded higher HRs (ranging from HR_BD_ = 3.49 [3.32–3.66] to HR_SZ_ = 12.70 [12.01–13.43]). Figure 1 shows the separation in HR between these classes of disorders. Within the ID group, the largest discrepancy between mild and severe ID was seen for AUD (HR_AUD_ = 5.63 [4.49–7.06]), and the lowest for BD (HR_BD_ = 1.50 [1.25–1.80]).

Next, we examined patterns of psychiatric diagnoses across the medical registries (Table 3) to identify those that appear in the PCR, are only in the PCR, and/or the inpatient and specialist registries. Among cases with ID, case registrations for internalizing disorders (MD, ANX, OCD, BD) were less likely to be found in the PCR or only in the PCR than G-Pop cases. Fewer than 100% of identified cases were found in the medical registries for ADHD, DUD, and AUD, possibly due to case registrations in the prescription drug and/or forensic registries. Among cases with ID, case registrations for psychotic disorders were slightly more likely to be in PCR and only PCR than the G-Pop population.

**Table 3.** Percentage of identified psychiatric cases that appear in the primary care, specialist, and inpatient registries for people with intellectual disability and the general population

	G-Pop: percent of cases that are				ID-Pop: percent of cases that are			
	Only in PCR^a^	In PCR	PCR + Specialist	PCR + Specialist + Inpatient	Only in PCR^a^	In PCR	PCR + Specialist	PCR + Specialist + Inpatient
MD	57.0%	83.7%	97.4%	100%	36.1%	67.9%	94.0%	100%
ANX	61.8%	85.8%	98.2%	100%	41.0%	73.5%	96.2%	100%
OCD	29.1%	57.4%	95.9%	100%	21.4%	50.3%	93.4%	100%
BD	8.5%	46.2%	92.9%	100%	8.9%	43.5%	85.9%	100%
ADHD	7.0%	45.3%	89.4%	91.1%	7.3%	48.0%	89.8%	93.8%
DUD	3.8%	19.8%	39.8%	50.4%	3.7%	21.5%	48.5%	60.0%
AUD	12.5%	37.2%	56.4%	71.5%	11.1%	37.8%	64.7%	82.0%
SZ	6.2%	45.1%	84.8%	100%	9.8%	48.9%	86.0%	100%
ONAP	7.3%	34.6%	76.9%	100%	10.9%	39.0%	80.5%	100%

Abbreviations: G-Pop = general population, ID = intellectual disability, PCR = primary care registry, MD = major depression, ANX = anxiety disorders, OCD = obsessive-compulsive disorder, BD = bipolar disorders, ADHD = attention-deficit/hyperactivity disorder, DUD = drug use disorder, AUD = alcohol use disorder, SZ = schizophrenia, ONAP = other non-affective psychoses.

a Only in PCR out of all medical registers (i.e. not found in specialist or inpatient).

The results of the cohort analyses show that the risk for all psychiatric disorders among people with ID compared to the G-Pop was significantly higher for all cohorts, but there is a general decreasing trend in the HR in most psychiatric diagnoses between the oldest (1958–1967) and the youngest (1988–1997) birth cohorts (see Table 4). In terms of magnitude from the oldest to the youngest cohort, the largest decreases in age-adjusted HR were observed for ONAP, OCD, and SZ. The trends for MD, ANX, OCD, and BD across cohorts were monotonic. However, DUD and AUD and SZ and ONAP exhibited a non-monotonic trend in which the magnitude of the HRs increased for the 1968–1977 birth cohort and decreased in each subsequent cohort. The HR for ADHD in people with ID compared to the G-Pop exhibited a small monotonic increase across birth cohorts. The linear trends on the effect of ID as a function of year of birth were statistically significant for all disorders except AUD.

**Table 4.** Hazard ratios with 95% confidence intervals of psychiatric risk for people with intellectual disabilities versus the general population across four birth cohorts

Birth Year	MD	ANX	OCD	BD	ADHD	DUD	AUD	SZ	ONAP
All	1.76 (1.72–1.79)^****^	1.88 (1.84–1.91)^****^	4.29 (4.11–4.49)^****^	3.49 (3.32–3.66)^****^	5.78 (5.65–5.91)^****^	1.72 (1.67–1.77)^****^	1.95 (1.88–2.02)^****^	12.70 (12.01–13.43)^****^	10.93 (10.56–11.32)^****^
1958–1967	2.18 (2.09–2.28)^****^	2.71 (2.59–2.84)^****^	9.61 (8.61–10.71)^****^	6.30 (5.76–6.88)^****^	4.83 (4.35–5.37)^****^	2.67 (2.46–2.90)^****^	1.77 (1.65–1.90)^****^	14.22 (13.07–15.47)^****^	13.94 (13.10–14.84)^****^
1968–1977	2.12 (2.03–2.22)^****^	2.44 (2.33–2.55)^****^	6.33 (5.69–7.05)^****^	4.64 (4.19–5.14)^****^	5.34 (4.94–5.78)^****^	2.90 (2.68–3.14)^****^	2.35 (2.18–2.54)^****^	14.42 (12.91–16.10)^****^	15.10 (14.08–16.20)^****^
1978–1987	1.68 (1.62–1.75)^****^	1.78 (1.72–1.85)^****^	3.91 (3.59–4.26)^****^	2.69 (2.43–2.97)^****^	5.69 (5.42–5.97)^****^	1.63 (1.53–1.73)^****^	2.02 (1.88–2.17)^****^	11.22 (9.89–12.73)^****^	9.83 (9.15–10.55)^****^
1988–1997	1.42 (1.37–1.46)^****^	1.45 (1.41–1.50)^****^	3.09 (2.88–3.32)^****^	2.27 (2.06–2.49)^****^	5.71 (5.55–5.87)^****^	1.38 (1.32–1.44)^****^	1.82 (1.70–1.96)^****^	8.09 (6.85–9.55)^****^	6.68 (6.21–7.20)^****^
Linear trend of year of birth^a^	0.86 (0.84–0.87)^****^	0.80 (0.79–0.82)^****^	0.68 (0.65–0.71)^****^	0.70 (0.67–0.73)^****^	1.04 (1.02–1.07)^**^	0.79 (0.77–0.81)^****^	0.99 (0.96–1.03)	0.87 (0.82–0.91)^****^	0.79 (0.77–0.82)^****^

*Note:* Models stratified by birth cohort, adjusted for sex, birth year, parents’ educational levels, and parents’ birth years, with interaction effects between age with the controlling variables. Significance levels for *p*-values: * < .05, ** < .01, *** < .001, **** < .0001.

Abbreviations: MD = major depression, ANX = anxiety disorders, OCD = obsessive-compulsive disorder, BD = bipolar disorders, ADHD = attention-deficit/hyperactivity disorder, DUD = drug use disorder, AUD = alcohol use disorder, SZ = schizophrenia, ONAP = other non-affective psychoses.

a The linear trend analysis includes an interaction term between intellectual disability and year of birth (measured on a time scale of 10 years), including all year of births in the same model.

## Discussion

This comprehensive national cohort study reveals dramatically elevated risks for psychiatric and substance use disorders among adults with ID compared to the general population, with particularly striking hazard ratios exceeding 10 for schizophrenia and other nonaffective psychoses. Notably, we also found significantly increased risks for substance use disorders, a finding less consistently reported in previous studies. Our inclusion of primary care data demonstrated its significant role in identifying psychiatric morbidity, including psychosis, within this population. Prior studies have examined only subsets of the population (e.g. those residing in certain counties or catchment areas (Lundqvist, 2013)) or accessed fewer registries (e.g. specialist and inpatient registries among those registered with the Swedish LSS (disability services) registry (Ahlström, Axmon, Sandberg, & Hultqvist, 2020; Axmon et al., 2018a)). We found that sizable portions of cases of psychiatric disorders in people with ID are only found in the primary care data, as has been found in the general population in Sweden (Sundquist et al., 2017) as well as surveys conducted by the World Health Organization for low-, middle-, and high-income countries (Wang et al., 2007). Similar patterns have been documented in UK primary care for adults with ID (Sheehan et al., 2015), suggesting that reliance on specialist-care registries alone may systematically underestimate psychiatric morbidity in people with ID across diverse healthcare systems. The identification and management of psychiatric conditions in primary care settings necessitates enhanced training for general practitioners and the development of consultation-liaison models that can support accurate diagnosis and appropriate treatment in community-based settings.

We also find that the proportion of MD and ANX cases (and OCD to a lesser extent) that are found in the primary care data for people with ID is 10–20 percentage points lower compared to the G-POP. However, the proportion of SZ and ONAP cases that are only found in the primary care data is larger by several percentage points for people with ID, although still only a minority of cases. It is possible that primary care physicians may have greater difficulty making differential diagnoses for patients with ID and are more likely to refer for further evaluation in specialty and inpatient settings, highlighting an area of need for future research and training.

The younger age distribution of individuals with ID in our sample, particularly those with mild ID (mean age 34.0 years versus 41.2 years in G-Pop), reflects both temporal changes in diagnostic practices and the relatively recent expansion of systematic ID identification in community and primary care settings. Nearly half (49.3%) of individuals with mild ID were aged 19–29 years at study end, compared to only 24.4% of the general population. This age differential was fully accounted for in our statistical models through adjustment for birth year and age interactions. The persistence of substantially elevated hazard ratios despite this conservative bias underscores the robustness of our findings and suggests that the true burden of psychiatric morbidity in people with ID may be even greater than our estimates indicate.

We stratified the prevalence of psychiatric disorders by level of ID, finding a general pattern wherein people with milder ID were at higher risk than people with more severe ID. These findings are consistent with some studies that have found a higher risk of psychiatric disorders among those with mild ID versus profound ID (Lund, 1985; Mazza et al., 2020), but inconsistent with other studies finding a higher risk among those with severe and profound ID (Cooper & Bailey, 2001; Cooper, Smiley, Morrison, Williamson et al., 2007). At face value, this finding may seem inconsistent with the neurodevelopmental spectrum hypothesis, which suggests that risk for ‘adult-onset’ disorders, such as BP, SZ, and ONAP, shares similar etiological pathways with other childhood-onset neurodevelopmental disorders such as ADHD and ID (Murray et al., 2017; Murray & Lewis, 1987; Owen et al., 2011). One might, therefore, predict that more severe ID would be associated with greater psychiatric risk. Previous work in the Swedish population revealed a monotonic negative association between measured IQ and SZ among males in the military conscription registry, which would have excluded those with ID (Kendler, Ohlsson, Sundquist, & Sundquist, 2015). Our findings suggest that the association between IQ and SZ may be more nuanced once the full population is included, including those with the lowest IQs, and may reflect the complexity of genetic, environmental, and neurodevelopmental pathways influencing both ID and risk for psychiatric conditions (Bergen et al., 2019; Drakesmith et al., 2019). Additionally, although individuals with mild ID are likely able to report symptoms of schizophrenia reliably (Meadows et al., 1991), challenges with the assessment and diagnosis of psychiatric symptoms among those at the most severe range of ID may also confound this association.

We also found that the magnitude of relative risk among people with ID for some psychiatric disorders (e.g. OCD, SZ, ONAP, ADHD) was substantially higher than the magnitude of relative risk for depression and anxiety, which is consistent with the neurodevelopmental spectrum and developmental risk factor models of these disorders (Murray et al., 2017; Murray & Lewis, 1987; Owen et al., 2011). Of note, anxiety and depression are common in the general population so that, on a relative scale, seeing the same magnitude of HRs for much rarer disorders, like SZ, would be unlikely. Were we to have used an additive scale, the largest ID-associated risk increases would be seen for ADHD, followed by anxiety and depression.

Together, these results present a complex picture within the neurodevelopmental spectrum framework: while the higher psychiatric risk observed in milder ID might appear counterintuitive to a simple monotonic relationship, this pattern could be influenced by diagnostic challenges in more severe ID (Meadows et al., 1991) and the intricate interplay of genetic and environmental factors (Bergen et al., 2019; Drakesmith et al., 2019). Conversely, the substantially elevated relative risks for disorders like SCZ, BP, and ADHD in the ID population align strongly with neurodevelopmental and developmental risk factor models (Murray et al., 2017; Murray & Lewis, 1987; Owen et al., 2011). This underscores the need for continued, nuanced longitudinal assessments of risk factors, symptoms, and timing across development to fully delineate the classification of these disorders within such a framework (Michelini et al., 2024).

The cohort analysis revealed that although the risk for psychiatric disorders among people with ID remained greater than the general population risk, the magnitude of difference decreased over time for almost all of the disorders. These findings are largely consistent with the geriatric Swedish cohorts in Axmon et al. (2018a). There were a few notable exceptions to this pattern. First, AUD and DUD exhibited a pattern in which the HR increased slightly for the 1968–1977 birth cohort compared to the 1958–1967 cohort and then decreased in each subsequent cohort (though remained greater than 1). It is interesting to note that these cohorts were born during the first major Swedish deinstitutionalization movement, which began with four major Acts of Parliament in 1954, 1967, 1985, and 1993 and the ultimate closing of institutions in 1999 (Tøssebro, 2016). The increased availability of home- and community-based services and increased social inclusion may have improved many mental health outcomes (Kim, Larson, & Charlie Lakin, 2001; Mansell, 2006; Young et al., 1998) but may also have increased access and social acceptance of substance use by persons with ID, particularly those with milder ID (Bhatt & Gentile, 2021; Huxley et al., 2019).

Another exception to the pattern of decreasing HRs across birth cohorts is that the HR for ADHD in people with ID increased slightly over time. This potentially reflects changing conceptualizations of the co-occurrence of ADHD (or hyperkinetic disorder -HKD) in the context of a developmental disability (Antshel et al., 2006). Previous versions of the DSM/ICD discouraged the diagnosis of ADHD/HKD in the presence of a developmental disability. More recent diagnostic criteria allow for the co-occurring diagnosis as long as symptoms are not fully explained by and are in excess of what would be expected given the level of developmental functioning.

## Limitations

Previous studies support the accuracy of the prevalence estimates of psychiatric disorders in the general population derived from the Swedish registry data (Ekholm et al., 2005; Kendler et al., 2018; Prescott & Kendler, 1999; Sellgren et al., 2011; Sundquist et al., 2017; Sundquist, Ohlsson, Sundquist, & Kendler, 2015). However, diagnosing psychiatric symptoms in people with limited cognitive or communication abilities can be challenging. Providers may differ in their assessment and diagnostic impressions (Cooper, Smiley, Morrison, Williamson et al., 2007), influenced by their training, experience with people with ID, prior knowledge of the prevalence of these conditions in people with ID, and reliance on diagnostic heuristics or confirmation bias (Kildahl, Oddli, & Helverschou, 2024). As previously noted (Kendler, Abrahamsson, Sundquist, & Sundquist, 2024), Swedish medical and criminal registries used to identify AUD and DUD may predominantly identify individuals with more severe manifestations of these disorders. These individuals are often included in the registry only if their alcohol-related problems lead to criminal justice encounters, such as repeated drunk driving, or significant medical interventions, thereby skewing prevalence estimates toward more severe cases. This method of identification contrasts with population-based personal interviews, which typically capture a broader spectrum of AUD severity. The markedly elevated rates of psychotic disorders imply that people with ID are more likely to be *diagnosed* with SZ or ONAP. Indeed, the extent to which people with ID are more prone to develop schizophrenia compared to the general population has been debated for nearly a century (Kendler & Zerbin-Rüdin, 1996; Turner, 1989). There may be systematic differences in the referral patterns for people with ID vs G-Pop with co-occurring psychiatric illness. Thus, studies are needed to examine the validity of registry diagnoses among people with ID compared to other diagnostic algorithms and the extent to which sources of diagnostic error vary for those with more severe ID.

## Conclusions

The results of this study demonstrate that people with ID are at increased risk for all of the psychiatric and substance disorders we examined compared to the general population with particularly striking hazard ratios exceeding 10-fold for psychotic disorders. These findings have direct implications for healthcare policy and service delivery. First, whole-system mental health screening protocols should be implemented as standard care for adults with ID, with particular attention to early detection of psychotic symptoms given the magnitude of risk observed. Second, primary care capacity-building is essential; our finding that substantial proportions of psychiatric cases in people with ID are identified solely in primary care settings (36–41% for depression and anxiety) indicates that general practitioners require enhanced training in recognizing and managing psychiatric symptoms in this population, potentially through consultation-liaison models with ID psychiatry specialists where available, or through structured diagnostic protocols where such specialists are absent (Moss et al., 1998; Sinclair et al., 2024). Third, targeted early intervention programs for psychotic disorders in people with ID should be developed and tested, learning from ultra-high-risk intervention models in the general population (Fusar-Poli et al., 2020), while adapting early psychosis assessment and treatment strategies for individuals at different severity levels of cognitive and communication limitations (Gómez, Navas, Verdugo, & Tassé, 2021). Fourth, service planners should allocate resources proportionate to the documented excess morbidity, recognizing that current mental health service utilization likely underestimates true need given diagnostic challenges in this population.

The narrowing gap in relative risks across birth cohorts may reflect improvements in social determinants (e.g. deinstitutionalization social inclusion, community supports, improved healthcare) and increased recognition of co-occurring psychiatric conditions (Fletcher, Barnhill, & Cooper, 2018). However, the persistently high relative risks, particularly the 10- to 12-fold elevation in risk for SZ and ONAP, demonstrate that substantial inequities remain. Investment in research on the etiology, diagnosis, prevention, and treatment of these conditions specific to people with ID is urgently needed to address this persistent health disparity.

## Supporting information

10.1017/S0033291725102882.sm001 Brown et al. supplementary material Brown et al. supplementary material

## Data Availability

Kristina Sundquist, MD, PhD had full access to all the data in the study and takes responsibility for the integrity of the data and the accuracy of the data analysis.

## Supplementary material

The supplementary material for this article can be found at https://doi.org/10.1017/S0033291725102882.

## Previous presentation

This paper has not previously been published or presented.

## Location of where work was done

Lund University, Virginia Commonwealth University.
