# Supplementary material for: Elevated psychiatric and substance use disorders in people with intellectual disabilities in a Swedish National Sample
Source: Psychol Med. 2025 Dec 29;56:e4. doi: 10.1017/S0033291725102882 (PMC12848947; doi:10.1017/S0033291725102882)
Supplement: Brown et al. supplementary material [file S0033291725102882sup001.docx]

Supplementary Material

Table S1 – Description of Registers

*Total Population Register*

The Total Population Register was created digitally in 1968 and includes yearly information, from the National Tax Board, on individuals registered in Sweden. It is possible to retrieve information on e.g. birth, death, immigration, emigration, migration within Sweden, place of residence, family information, civil status, etc. For more information, see [*https://www.scb.se/contentassets/8f66bcf5abc34d0b98afa4fcbfc0e060/rtb-bar-2016-eng.pdf*](https://www.scb.se/contentassets/8f66bcf5abc34d0b98afa4fcbfc0e060/rtb-bar-2016-eng.pdf)

*Multi-Generation Register*

The Multi-Generation Register is a register made up of persons who have been registered in Sweden at some time since 1961 and those who were born in 1932 or later. These are called index persons. The register contains connections between index persons and their biological parents. In 2016, about 12 million index persons were included in the register. The Multi-Generation Register is a part of the register system for Total Population Register, where information comes from the National Tax Board. Every year, a new version of the register is created, including new index persons who immigrated or were born during the year. Information from the Multi-Generation Register may be disclosed for research and statistical purposes. See *https://www.scb.se/vara-tjanster/bestall-data-och-statistik/register/flergenerationsregistret/*

*National Patient Register*

In the 1960's the National Board of Health and Welfare started to collect information regarding in-patients at public hospitals, the National Patient Register (NPR). Initially it contained information about all patients treated in psychiatric care and approximately 16 percent of patients in somatic care. The register at that time covered six of the 26 county councils in Sweden. In 1984, the Ministry of Health and Welfare together with the Federation of County Councils decided a mandatory participation for all county councils. From 1987, NPR includes all in-patient care in Sweden. Since 2001, the register also covers outpatient doctor visits including day surgery and psychiatric care from both private and public caregivers. For more information, see *https://www.socialstyrelsen.se/en/statistics-and-data/registers/national-patient-register/*

*Primary Care Data*

We also used information from our new Primary Care research dataset including individual-level information on clinical diagnoses from primary health care centers from the following Swedish counties: Blekinge (2009-2018), Dalarna (2005-2018), Gotland (2011-2018), Gävleborg (2010-2018), Halland (2007-2018), Jönköping (2008-2018), Kalmar (2007-2018), Kronoberg (2006-2018), Norrbotten (2001-2018), Skåne (1989-2018), Stockholm (2003-2018), Södermanland (1992-2018), Uppsala (2005-2018), Västra Götaland (2000-2018), Värmland (2005-2018), Västerbotten (1991-2018), Västernorrland (2008-2018), Västmanland (2014-2018), Östergötland (1990-2018), and Örebro (2006-2018). The retrieval of data differs due to timing of digitalization of patient records. In 2018, 99% of the Swedish population lived in these 20 counties. For more information see *Sundquist, J., Ohlsson, H., Sundquist, K. et al. Common adult psychiatric disorders in Swedish primary care where most mental health patients are treated. BMC Psychiatry 17, 235 (2017).*

*The Population and Housing Censuses*

Every fifth year between 1960 and 1990 Sweden conducted censuses. These registers include among other things, the population's employment, educational level, the composition of households and housing. See *https://www.scb.se/hitta-statistik/statistik-efter-amne/befolkning/befolkningens-storlek-och-forandringar/registerbaserad-folk--och-bostadsrakning-census/*

*Longitudinal integrated database for health insurance and labour market studies (LISA)*

LISA was created by Statistics Sweden in 1990 and includes yearly information on e.g. employment and educational level on individuals (from age 16) registered in Sweden. See *https://www.scb.se/en/services/ordering-data-and-statistics/register/longitudinal-integrated-database-for-health-insurance-and-labour-market-studies-lisa/*

*Prescribed Drug Register*

The Swedish Prescribed Drug Register started in July 2005 and includes all prescribed drugs being fetched at pharmacies, linked to personal numbers. See *https://www.socialstyrelsen.se/en/statistics-and-data/registers/national-prescribed-drug-register/*

*Cause of Death Register*

The Cause of Death Register includes all deaths occurring in Sweden from 1961 (including for Swedish citizens dying abroad) and is updated yearly. There is also a historical register between the years 1952 to 1960. For more information, see *https://www.socialstyrelsen.se/en/statistics-and-data/registers/national-cause-of-death--register/*

*Criminal and Suspicion Register*

The Swedish Criminal Register and the Swedish Suspicion Register includes individual-level information on all committed crimes from 1973 and all suspicions of crimes related to an individual from 1998. For more information, see *https://polisen.se/lagar-och-regler/behandling-av-personuppgifter/polisens-register/*

Table S2 - Definition of disorders and variables

| Disorder/Variable | Registers Used | Definition |
| --- | --- | --- |
| Intellectual Disability  (ID) | Hospital Discharge Register;  Outpatient Care Register;  Primary Care Data | Intellectual Disability (ID) was identified in the Swedish medical registries by ICD codes: ICD9: 317, 318A, 318B, 318C, 319; ICD10: F70, F71, F72, F73, F78 and F79. |
| Mild/Moderate/Severe Intellectual Disability | Hospital Discharge Register;  Outpatient Care Register;  Primary Care Data | Mild, Moderate and Severe Intellectual Disability were identified in the Swedish medical registries by codes: ICD9: 317 and ICD10: F70, ICD9: 318A and ICD10: F71, and ICD9: 318B, 318C and ICD10: F72 and F73, respectively. Severe ID was defined as ICD10: F72 and F73 and ICD9: 318B, 318C; moderate ID was defined as ICD10: F71 and ICD9: 318A, without any occurrence of severe ID; mild ID was defined as ICD10: F70 and ICD9: 317, without any occurrence of severe or moderate ID. |
| Major Depression  (MD) | Hospital Discharge Register;  Outpatient Care Register;  Primary Care Data | Major Depression (MD) was identified in the Swedish medical registries by ICD codes: ICD8: 296.0, 296.2, 298.0, 300.4; ICD9: 296B, 298A, 300E; ICD10: F32, F33. |
| Anxiety Disorder  (AD) | Hospital Discharge Register;  Outpatient Care Register;  Primary Care Data | Anxiety Disorder (AD) was identified in the Swedish medical registries by ICD codes: ICD-8: 300.0, 300.2; ICD-9: 300A, 300C; ICD-10: F40, F41 (except F41.2). |
| Obsessive-Compulsive Disorder  (OCD) | Hospital Discharge Register;  Outpatient Care Register;  Primary Care Data | Obsessive-Compulsive Disorder (OCD) was identified in the Swedish medical registries by ICD codes: ICD8: 300.3; ICD9: 300D; ICD10: F42. |
| Bipolar Disorder  (BD) | Hospital Discharge Register;  Outpatient Care Register;  Primary Care Data | Bipolar Disorder (BD) was identified in the Swedish medical registries by ICD codes: ICD8: 296.1, 296.3, 296.8, 296.9, 298.1; ICD9: 296A, 296C, 296D, 296E, 296W, 298B; ICD10: F30, F31. |
| Attention Deficit Hyperactivity Disorder  (ADHD) | Hospital Discharge Register;  Outpatient Care Register;  Primary Care Data;  Prescribed Drug Register | Attention Deficit Hyperactivity Disorder (ADHD) was identified in the Swedish medical registries by ICD codes: ICD9: 314; ICD10: F90; in the Prescribed Drug Register by the drugs amfetamine (Anatomical Therapeutic Chemical (ATC) Classification System N06BA01), dexamfetamine (N06BA02), methylphenidate (N06BA04), and atomoxetine (N06BA09). |
| Drug Use Disorder  (DUD) | Hospital Discharge Register;  Outpatient Care Register;  Primary Care Data;  Prescribed Drug Register;  Cause of Death Register;  Criminal Register;  Suspicion Register | DUD was identified in the Swedish medical and mortality registries by ICD codes (ICD8: Drug dependence (304); ICD9: Drug psychoses (292), Drug dependence (304) and Nondependent abuse of drugs of type Sedatives, Opioids or Amphetamine (305E, 305F, 305H); ICD10: Mental and behavioral disorders due to psychoactive substance use (F10-F19), except those due to alcohol (F10) or tobacco (F17)); in the Suspicion Register by codes 3070, 5010, 5011, and 5012, that reflect crimes related to DUD; and in the Crime Register by references to laws covering narcotics (law 1968:64, paragraph 1, point 6) and drug-related driving offences (law 1951:649, paragraph 4, subsection 2 and paragraph 4A, subsection 2). DUD was identified in individuals (excluding those suffering from cancer) in the Prescribed Drug Register who had retrieved (in average) more than four defined daily doses a day for 12 months from either of Hypnotics and Sedatives (Anatomical Therapeutic Chemical (ATC) Classification System N05C and N05BA), Opioids (ATC: N02A) or Methadone (ATC: N07BC). |
| Alcohol Use Disorder  (AUD) | Hospital Discharge Register;  Outpatient Care Register;  Primary Care Data;  Prescribed Drug Register;  Cause of Death Register;  Criminal Register;  Suspicion Register | Alcohol Use Disorder (AUD) was identified in the Swedish medical and mortality registries by ICD codes: ICD8: 571.0, 291, 303; ICD9: V79B, 305A, 357F, 571A-D, 425F, 535D, 291, 303; ICD 10: E24.4, G31.2, G62.1, G72.1, I42.6, K29.2, K70, K85.2, K86.0, O35.4, F10.1-F10.9; in the Swedish Criminal Register and the Swedish Suspicion Register with at least two registrations of drunk driving (suspicion code 3005, law 1951:649 (paragraph 4 and 4A)) or drunk in charge of a maritime vessel (suspicion code 3201, law 1994:1009 (chapter 20, paragraph 4 and 5)); in the Prescribed Drug Register by the drugs disulfiram (Anatomical Therapeutic Chemical (ATC) Classification System N07BB01), acamprosate (N07BB03), and naltrexone (N07BB04). |
| Schizophrenia  (SZ) | Hospital Discharge Register;  Outpatient Care Register;  Primary Care Data | Schizophrenia (SZ) was identified in the Swedish medical registries by ICD codes: ICD8: 295.1, 295.2, 295.3, 295.6, 295.9; ICD9: 295B, 295C, 295D, 295G, 295X; ICD10: F20.0, F20.1, F20.2, F20.3, F20.5, F20.9. |
| Other Non-affective Psychosis (ONAP) | Hospital Discharge Register;  Outpatient Care Register;  Primary Care Data | Other Non-affective Psychosis (ONAP) was identified in the Swedish medical registries by ICD codes: ICD8: 297, 298.3, 298.9, 295.4, 295.7; ICD9: 297, 298E, 298W, 298X, 295E, 295H, 295W; ICD10: F22, F23, F24, F25, F26, F27, F28, F29, F208. |

Table S3 – Details on R-packages used in statistical analyses

1. Therneau T. survival: A package for Survival Analysis in R. R package. 2024.
2. Wickham H. ggplot2: Elegant Graphics for Data Analysis. New York, NY: Springer-Verlag; 2016.
3. Wickham H, Miller E, Smith D. haven: Import and Export 'SPSS', 'Stata' and 'SAS' Files. R package. 2023.
4. Wickham H, François R, Henry L, Müller K, Vaughan D. dplyr: A Grammar of Data Manipulation. R package. 2023.
5. Barrett T, Dowle M, Srinivasan A, Gorecki J, Chirico M, Hocking T. data.table: Extension of ‘data.frame’. R package. 2024.
6. Kassambara A, Kosinski M, Biecek P. survminer: Drawing Survival Curves using ‘ggplot2’. R package. 2021.
7. Nightingale Health Ltd. ggforestplot. R package. 2020. Github Repository: https://github.com/NightingaleHealth/ggforestplot
8. Wickham H, Averick M, Bryan J, Chang W, McGowan LD, François R, Grolemund G, Hayes A, Henry L, Hester J, Kuhn M, Pedersen TL, Miller E, Bache SM, Müller K, Ooms J, Robinson D, Seidel DP, Spinu V, Takahashi K, Vaughan D, Wilke C, Woo K, Yutani H. Welcome to tidyverse. *Journal of Open Source Software*, 4(43), 1686.
